# Open questions on water confined in nanoporous materials

**DOI:** 10.1038/s42004-021-00544-9

**Published:** 2021-07-14

**Authors:** François-Xavier Coudert, Anne Boutin, Alain H. Fuchs

**Affiliations:** 1 Chimie ParisTech, PSL University, CNRS, Institut de Recherche de Chimie Paris, Paris, France; 2 PASTEUR, Département de Chimie, École Normale Supérieure, PSL University, Sorbonne Université, CNRS, 75005 Paris, France

**Keywords:** Physical chemistry, Materials chemistry

## Abstract

Water adsorption in soft nanoporous materials can trigger large-scale structural transitions and introduce new properties in the confined water phase. Here, we look at some of the outstanding questions in this lively field of research.

The understanding of the impact of confinement at the molecular (or nanometric) scale on the properties of polar fluids, and of water, in particular, is an open physico-chemical problem of both practical and fundamental interest. Practical, because residual water plays an important role in many industrial processes, such as separation and catalysis, or biological systems; and fundamental, because the behavior of water confined by complex and heterogeneous surfaces is still to be rationalized. Water is a strongly polar molecule, and as such its adsorption into nanoporous materials gives rise to a rich phenomenology due to strong guest–guest interactions, and tunable host–guest interactions, depending on the chemical nature of the nanopores surface. Based on the balance between these factors, adsorption of water can take place in the vapor or the liquid phase, i.e., with a gamut of characteristic pressure that goes from Pa to the GPa^1^. Moreover, water–water interactions are strongly directional and forming a characteristic hydrogen bond network. This leads to structural and dynamic frustration arising in the confined liquid phase, due to the external confining field imposed by the host framework.

In order to probe the rich behavior of these complex systems, researchers in the last decade have used an increasing number of techniques, both experimental and computational, leading to rapid progress in our understanding of the structure, dynamics, and thermodynamics of water in confined spaces. In particular, there has been increased adoption of in situ setups with varying temperature and pressure (or water content), including X-ray and neutron scattering, vibrational spectroscopies (infrared, Raman, and sum-frequency generation), and NMR. In addition, many large-scale studies now routinely include molecular simulation at different levels of description: from Monte Carlo simulations, classical molecular dynamics, to ab initio methods (e.g., first-principles molecular dynamics) and even path integral simulations, which take into account the quantum nature of nuclei.

Despite the availability of such advanced tools, there are many open questions about the nature, behavior, and properties of water confined in structurally and chemically complex nanopores. We note here that the unusual properties of water under confinement in dynamic spaces has been long recognized in the study of biomolecules, where proteins exploit some of the anomalous properties of confined water in their biological function (e.g., to ensure rapid water flow in aquaporins or to gate proton flow in proton pumps and enzymes)^2,3^. In this piece, we provide a short introduction to the recent progress and open questions in the behavior of water confined inside materials at the nanoscale.

## Beyond the “rigid host” approximation

One area that has seen rapid development is that of water confinement in highly flexible nanoporous materials. The past two decades have seen the multiplication of novel nanoporous materials based on molecular or supramolecular frameworks, such as metal–organic frameworks (MOFs), covalent organic frameworks, and supramolecular organic frameworks. Compared to inorganic materials, such as zeolites, this new generation of nanoporous solids—named “soft porous crystals”—are based on relatively weak supramolecular interactions and demonstrate large-scale intrinsic structural flexibility. In order to fully understand the behavior of water in these dynamic materials, we need to shift our focus from the classical view (or approximation) of the “rigid host” material as an inert matrix, that acts merely as a fixed external potential on the adsorbed phase.

One of the areas where the effect of the host flexibility is perhaps the strongest, and not yet fully understood, is in the intrusion of water at high pressure in hydrophobic soft porous crystals^4,5^. There, two different responses are intertwined: the response of the host framework to the hydrostatic pressure exerted, and the deformation linked to the presence of the adsorbate inside the pores—which exerts anisotropic adsorption stress, from within the pores^6^. This two-pronged effect has been reported in the past in several MOFs and some zeolites (although with a smaller amplitude of strain) and is sometimes referred to as “superhydration”, because of the larger water uptake that is made possible by the flexibility of the host.

## Water adsorption introducing new properties

**Fig. 1:**
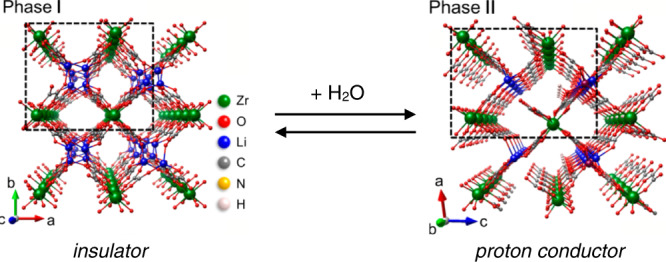
Structural change of a metal–organic framework upon water adsorption. A proton-conducting phase, owing to a hydrogen-bonded chain of water molecules, forms in the pores of the MOF ((CH_3_)_2_NH_2_)_2_[Li_2_Zr(C_2_O_4_)_4_] upon water adsorption. Reprinted with permission from ref. 8. Copyright 2015 American Chemical Society.

In some cases, the adsorption of water in flexible structures can lead to a structural transition into a phase with novel and generally rare properties for crystalline materials. For example, hydration near water saturation pressure can lead to the emergence of a superprotonic phase in a zirconium phosphonate MOF, with very high proton conductivity^7^. A similar effect was observed in ((CH_3_)_2_NH_2_)_2_[Li_2_Zr(C_2_O_4_)_4_], as depicted in Fig. 1: it exemplifies well how the structural changes triggered by water adsorption introduce new properties in the confined water, that does not derive from either the parent framework or the bulk water^8^.

Another case of interest, which has only recently been evidenced and understood, is that of “negative hydration expansion”, i.e., the occurrence of strong contraction of porous materials upon water adsorption. Indeed, while adsorption of guest molecules is generally considered to lead to material expansion and stiffening, in some specific cases the inverse effect is possible: pore shrinking and softening is a counterintuitive mechanical response^9^. This phenomenon is most often observed in soft materials, but it can also occur in more rigid frameworks if the host–guest interactions are particularly strong—as water–host interactions can be. One such example is the contraction (10% volume shrinkage) of the oxide ZrW_2_O_8_ under hydration, a “cold case” of old experimental findings that was only recently solved by X-ray diffraction, total scattering, and quantum mechanical calculations^10^.

It was recently proposed and demonstrated that this “negative hydration expansion” could be leveraged into the fabrication of actuators and metamaterials^11^. While water itself is known for its many anomalous physical properties, it is interesting to think of how it can also impart new properties and new functions to porous materials.

## Reactivity of confined water

Like other chemical properties, molecular reactivity is strongly affected by confinement at the molecular scale. This effect is at play in heterogeneous catalysts and enzymes^12^, and can even be used to control reactivity quantitatively through hybrid light–matter states^13^. However, there remain many open questions about the impact of confinement on liquid water. One recent example showing the vast prospects opened by this effect are the recent studies demonstrating that confinement of water in ionic and organic solvents can control its reactivity at electrified interfaces^14^. Changing the composition of these electrolytes of water confined in an acetonitrile matrix leads to changes in water–ion coordination and water adsorption at the electrode interface, therefore, allowing fine-tuning of the reactivity of the system, controlling the electric potential at which the hydrogen evolution reaction occurs

Another example of the need to take into account water reactivity in complex chemical systems is the study of water confined between graphene oxide (GO) layers. GO is a graphene-based material presenting a high number of disordered oxygen-bearing groups (hydroxyl, epoxides, and carboxylic acids). Recent ab initio molecular dynamics simulations of chemically realistic models of GO have confirmed that chemical processes occur at the GO/liquid water interface, giving rise to a negative charge of the GO layers, drastically affecting the behavior of water near the interface by disrupting its hydrogen-bonding patterns and speeding up its transport^15^.

These questions of reactivity of confined water are directly related to the water stability of supramolecular porous frameworks. Several applications (including heterogeneous catalysis, water harvesting from air, and energy storage) require porous frameworks that are hydrothermally and chemically stable, yet many MOFs have been demonstrated to be unstable in the presence of water, depending on the temperature or pH^16^. Even for materials that are considered structurally stable, the impact of aging in presence of water is typically not well characterized, and still poorly understood.

## Conclusions

In conclusion, we observe a growing need for the characterization of confined water and its interactions with nanoporous materials, at multiple scales of length and time, through in situ and *in operando* experimental techniques as well as multiscale computational approaches. Such synergistic studies allow for the description of more complex phenomena, including structure, thermodynamics and chemical reactivity, in nanoporous materials whose complexity—both in terms of structure and behavior—are becoming ever more sophisticated. This is particularly true for flexible materials, with chemically heterogeneous pore surfaces, oftentimes embedded into hierarchical or nanostructured composite materials.

## References

[1] Fraux G, Coudert F-X, Boutin A, Fuchs AH (2017). Forced intrusion of water and aqueous solutions in microporous materials: from fundamental thermodynamics to energy storage devices. Chem. Soc. Rev..

[2] Rasaiah JC, Garde S, Hummer G (2008). Water in nonpolar confinement: from nanotubes to proteins and beyond. Annu. Rev. Phys. Chem..

[3] Lynch CI, Rao S, Sansom MSP (2020). Water in nanopores and biological channels: a molecular simulation perspective. Chem. Rev..

[4] Ortiz G, Nouali H, Marichal C, Chaplais G, Patarin J (2014). Versatile energetic behavior of ZIF-8 upon high pressure intrusion–extrusion of aqueous electrolyte solutions. J. Phys. Chem. C.

[5] Michelin-Jamois M, Picard C, Vigier G, Charlaix E (2015). Giant osmotic pressure in the forced wetting of hydrophobic nanopores. Phys. Rev. Lett..

[6] Neimark AV, Coudert F-X, Boutin A, Fuchs AH (2009). Stress-based model for the breathing of metal-organic frameworks. J. Phys. Chem. Lett..

[7] Hassanzadeh Fard Z (2018). Superprotonic phase change to a robust phosphonate metal–organic framework. Chem. Mater..

[8] Tominaka S, Coudert F-X, Dao TD, Nagao T, Cheetham AK (2016). Insulator-to-proton-conductor transition in a dense metal–organic framework. J. Am. Chem. Soc..

[9] Mouhat F (2015). Softening upon adsorption in microporous materials: a counterintuitive mechanical response. J. Phys. Chem. Lett..

[10] Baise M (2018). Negative hydration expansion in ZrW_2_O_8_: microscopic mechanism, spaghetti dynamics, and negative thermal expansion. Phys. Rev. Lett..

[11] Wei Y-L, Yang Q-S, Ma L-H, Tao R, Shang J-J (2020). Design and analysis of 2D/3D negative hydration expansion Metamaterial driven by hydrogel. Mater. Des..

[12] Küchler A, Yoshimoto M, Luginbühl S, Mavelli F, Walde P (2016). Enzymatic reactions in confined environments. Nat. Nanotechnol..

[13] Ebbesen TW (2016). Hybrid light–matter states in a molecular and material science perspective. Acc. Chem. Res..

[14] Serva A, Dubouis N, Grimaud A, Salanne M (2021). Confining water in ionic and organic solvents to tune its adsorption and reactivity at electrified interfaces. Acc. Chem. Res..

[15] Mouhat F, Coudert F-X, Bocquet M-L (2020). Structure and chemistry of graphene oxide in liquid water from first principles. Nat. Commun..

[16] Wang C, Liu X, Keser Demir N, Chen JP, Li K (2016). Applications of water stable metal–organic frameworks. Chem. Soc. Rev..

